# Ethnic differences in the effects of apolipoprotein E ɛ4 and vascular risk factors on accelerated brain aging

**DOI:** 10.1093/braincomms/fcae213

**Published:** 2024-07-12

**Authors:** Yanghee Im, Sung Hoon Kang, Gilsoon Park, Heejin Yoo, Min Young Chun, Chi-Hun Kim, Chae Jung Park, Jun Pyo Kim, Hyemin Jang, Hee Jin Kim, Kyungmi Oh, Seong-Beom Koh, Jong-Min Lee, Duk L Na, Sang Won Seo, Hosung Kim

**Affiliations:** USC Steven Neuroimaging and Informatics Institute, Keck School of Medicine of University of Southern California, Los Angeles, CA 90033, USA; Department of Biomedical Engineering, Hanyang University, Seoul 04763, Korea; Department of Neurology, Samsung Medical Center, Sungkyunkwan University School of Medicine, Seoul 06351, Korea; Department of Neurology, Korea University Guro Hospital, Korea University College of Medicine, Seoul 08308, Korea; USC Steven Neuroimaging and Informatics Institute, Keck School of Medicine of University of Southern California, Los Angeles, CA 90033, USA; Department of Neurology, Samsung Medical Center, Sungkyunkwan University School of Medicine, Seoul 06351, Korea; Department of Neurology, Yongin Severance Hospital, Yonsei University College of Medicine, Yongin 16995, Korea; Department of Neurology, Hallym University Sacred Heart Hospital, Hallym University College of Medicine, Anyang 14068, Korea; Research Institute, National Cancer Center, Goyang 10408, Korea; Department of Neurology, Samsung Medical Center, Sungkyunkwan University School of Medicine, Seoul 06351, Korea; Department of Neurology, Samsung Medical Center, Sungkyunkwan University School of Medicine, Seoul 06351, Korea; Department of Neurology, Samsung Medical Center, Sungkyunkwan University School of Medicine, Seoul 06351, Korea; Department of Neurology, Korea University Guro Hospital, Korea University College of Medicine, Seoul 08308, Korea; Department of Neurology, Korea University Guro Hospital, Korea University College of Medicine, Seoul 08308, Korea; Department of Biomedical Engineering, Hanyang University, Seoul 04763, Korea; Department of Neurology, Samsung Medical Center, Sungkyunkwan University School of Medicine, Seoul 06351, Korea; Department of Neurology, Samsung Medical Center, Sungkyunkwan University School of Medicine, Seoul 06351, Korea; Department of Digital Health, SAIHST, Sungkyunkwan University, Seoul 06355, Korea; Department of Health Sciences and Technology, SAIHST, Sungkyunkwan University, Seoul 06355, Korea; Alzheimer’s Disease Convergence Research Center, Samsung Medical Center, Seoul 06351, Korea; Department of Intelligent Precision Healthcare Convergence, Sungkyunkwan University, Suwon 16419, Korea; USC Steven Neuroimaging and Informatics Institute, Keck School of Medicine of University of Southern California, Los Angeles, CA 90033, USA

**Keywords:** *APOE* ɛ4, vascular risk factors, ethnicity, brain age

## Abstract

The frequency of the apolipoprotein E ɛ4 allele and vascular risk factors differs among ethnic groups. We aimed to assess the combined effects of apolipoprotein E ɛ4 and vascular risk factors on brain age in Korean and UK cognitively unimpaired populations. We also aimed to determine the differences in the combined effects between the two populations. We enrolled 2314 cognitively unimpaired individuals aged ≥45 years from Korea and 6942 cognitively unimpaired individuals from the UK, who were matched using propensity scores. Brain age was defined using the brain age index. The apolipoprotein E genotype (ɛ4 carriers, ɛ2 carriers and ɛ3/ɛ3 homozygotes) and vascular risk factors (age, hypertension and diabetes) were considered predictors. Apolipoprotein E ɛ4 carriers in the Korean (*β* = 0.511, *P* = 0.012) and UK (*β* = 0.302, *P* = 0.006) groups had higher brain age index values. The adverse effects of the apolipoprotein E genotype on brain age index values increased with age in the Korean group alone (ɛ2 carriers × age, *β* = 0.085, *P* = 0.009; ɛ4 carriers × age, *β* = 0.100, *P* < 0.001). The apolipoprotein E genotype, age and ethnicity showed a three-way interaction with the brain age index (ɛ2 carriers × age × ethnicity, *β* = 0.091, *P* = 0.022; ɛ4 carriers × age × ethnicity, *β* = 0.093, *P* = 0.003). The effects of apolipoprotein E on the brain age index values were more pronounced in individuals with hypertension in the Korean group alone (ɛ4 carriers × hypertension, *β* = 0.777, *P* = 0.038). The apolipoprotein E genotype, age and ethnicity showed a three-way interaction with the brain age index (ɛ4 carriers × hypertension × ethnicity, *β*=1.091, *P* = 0.014). We highlight the ethnic differences in the combined effects of the apolipoprotein E ɛ4 genotype and vascular risk factors on accelerated brain age. These findings emphasize the need for ethnicity-specific strategies to mitigate apolipoprotein E ɛ4-related brain aging in cognitively unimpaired individuals.

## Teaser

Im *et al*. reported that the combined effects of apolipoprotein E ɛ4 and vascular risk factors, particularly age and hypertension, have a stronger impact on the brain age index in the Korean group compared to the UK group. These findings underscore the need for ethnicity-specific strategies to manage vascular risk factors to mitigate apolipoprotein E ɛ4-related brain aging.

## Graphical Abstract

**Graphical Abstract:**
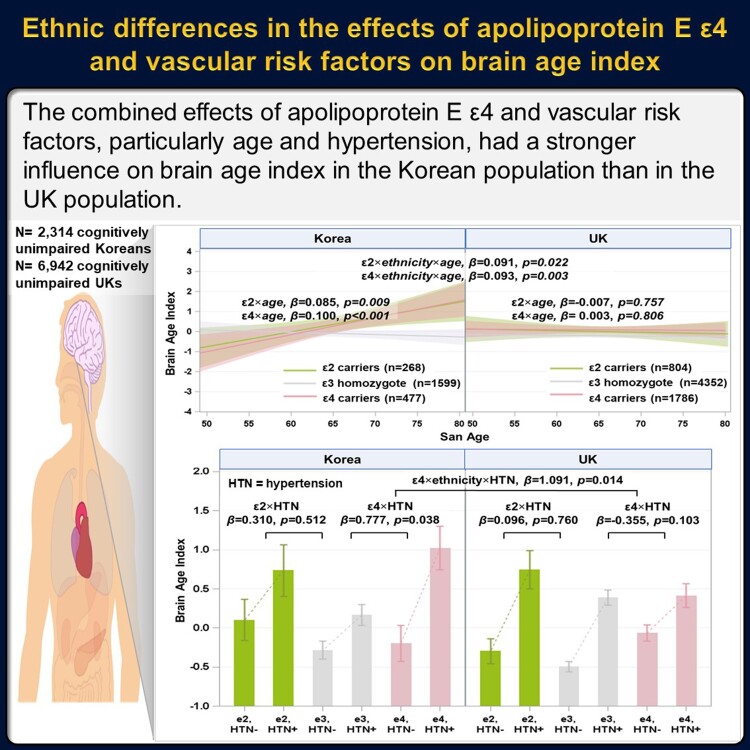

## Introduction

Apolipoprotein E (*APOE*) ɛ4 allele is the most influential genetic risk factor for developing Alzheimer’s disease.^1^ A growing body of evidence suggests that *APOE* ɛ4 carriers experience accelerated brain atrophy, even in the absence of cognitive impairment.^1^ This atrophy predominantly affects regions associated with Alzheimer’s disease, such as the medial temporal and parietal regions.^1-3^ Additionally, vascular risk factors, including age, hypertension and diabetes, have detrimental effects on brain atrophy, primarily in the frontal and temporal regions.^4-6^ Importantly, *APOE* ɛ4 and vascular risk factors synergistically contribute to cognitive decline.^7-9^ Notably, the combined effects of *APOE* ɛ4 and hypertension^7^ and *APOE* ɛ4 and diabetes^8^ on cognitive decline are observed in cognitively unimpaired (CU) individuals.

*APOE* ɛ2 allele is a protective factor against developing Alzheimer’s disease.^10,11^ However, due to the low proportion of *APOE* ɛ2, studies investigating the association between *APOE* ɛ2 and brain atrophy are relatively sparse. Consequently, these associations remain controversial. Previous studies on the impacts of *APOE* ɛ2 on brain atrophy have linked ɛ2 carriers to relatively less brain atrophy in medial temporal regions.^12,13^ In contrast, several studies have shown the opposite results that ɛ2 carriers are associated with brain atrophy in the hippocampus.^14,15^

The trajectory of brain atrophy throughout aging can be captured and translated into an individual's brain age using machine learning algorithms.^16^ Brain age serves as an indicator of the overall brain health as it allows individual-level inferences rather than group-level assessments. Previous studies have linked vascular risk factors, such as hypertension, diabetes and obesity, to accelerated brain aging in CU populations.^17,18^ Studies conducted in European and American populations have explored the relationship between *APOE* ɛ4 and brain age and reported the adverse effects of *APOE* ɛ4 on brain age.^19,20^ However, there is currently a gap in the literature regarding the occurrence of these effects in Asian populations and whether the impact of *APOE* ɛ4 differs among various racial and ethnic groups.

The proportion of *APOE* ɛ4 carriers is lower in the Asian population than in the European population.^21-24^ Nonetheless, the impact of *APOE* ɛ4 on dementia development is more pronounced in the Asian population.^25-27^ Conversely, vascular risk factors and their associated complications are more prevalent in Asian populations than in European populations.^28,29^ Our recent study indicated that ethnicity modulates the effects of vascular risk factors on brain age,^30^ with a greater impact observed in the Korean population than in the UK population.^30^ Given the differences in the frequency of *APOE* ɛ4 carriers, vascular risk factors and their influence on brain health among ethnic groups, we hypothesized that there may be disparities in the combined effects of *APOE* ɛ4 and vascular risk factors on brain age across ethnic groups.

In this study, we aimed to investigate the relationships between *APOE* ɛ4, *APOE* ɛ2, vascular risk factors and brain age measured using the brain age index (BAI) in Korean and UK CU individuals. First, we examined whether *APOE* ɛ4 or ɛ2 increased the BAI values in both populations. Second, we assessed the combined effects of *APOE* ɛ4 or ɛ2 and vascular risk factors on the BAI values in both populations. Finally, we determined the potential differences in these combined effects between the two populations.

## Materials and methods

### Study populations

Individuals aged ≥45 years were recruited from the Health Promotion Center at the Samsung Medical Center (SMC-HPC, Seoul, Korea) and underwent a comprehensive battery of health screening examinations between 1 September 2008 and 31 October 2019. A total of 4782 eligible candidates identified as CU underwent a full medical examination comprising cognitive assessment, *APOE* genotyping and 3.0-T MRI examination, including a high-resolution T_1_-weighted MRI examination, as part of the standard screening for dementia. Participants were excluded if they met the following criteria: 728 had missing data on years of education or Mini-Mental State Examination score^31^; 509 showed significant cognitive impairment determined either by a Mini-Mental State Examination score below the 16th percentile in age-, sex- and education-matched norms or through an interview with a qualified neurologist; 312 exhibited severe cerebral white matter hyperintensities (deep white matter ≥ 2.5 cm and caps or band ≥ 1.0 cm) or structural brain lesions, such as territorial infarction, lobar hemorrhage, brain tumor and hydrocephalus; 542 had missing data on DM, hypertension or body mass index; and 377 had unreliable cortical thickness analysis due to head motion, blurred MRI, improper registration to a standardized stereotaxic space, misclassification of tissue type or imprecise surface extraction. Finally, 2314 participants were included in this study.

Similarly, data from individuals of British ancestry were sourced from the UK Biobank (UKB, http://www.ukbiobank.ac.uk). Only white adults, such as British (93.7%), Irish (2.77%) and other backgrounds (3.44%), were included in the study. Additionally, we excluded subjects with a self-reported or hospital record-based history of dementia, Parkinson's disease or other central nervous system disorders were excluded. Finally, 17 340 CU individuals registered in the UKB were included after applying the inclusion and exclusion criteria, alongside the random selection of participants for brain imaging data processing.

Propensity score matching was performed using a multivariate logistic regression analysis based on age and sex. A total of 2314 Korean individuals were paired with 6942 UK individuals through propensity score matching using the 1:3 nearest-neighbor algorithm with a caliper of 0.25.

This study received approval from the Institutional Review Board of the Samsung Medical Center and followed the ethical guidelines outlined in the Declaration of Helsinki. All participants recruited from the SMC-HPC provided written informed consent. Anonymous and de-identified data from the UKB were utilized for analysis, thereby exempting the present study from obtaining informed consent.

### *APOE* genotyping

For participants from the SMC, genomic DNA was extracted from peripheral blood leukocytes using the Wizard Genomic DNA Purification Kit, following the manufacturer's protocol (Promega, Madison, WI, USA). Genotyping of the single nucleotide polymorphisms within the *APOE* gene (rs429358 in codon 112 and rs7412 in codon 158) was conducted using TaqMan Single Nucleotide Polymorphism Genotyping Assay (Applied Biosystems, Foster City, CA, USA) on a 7500 Fast Real-Time PCR System (Applied Biosystems), following the manufacturer's instructions. For participants from the UKB, genotype calling was carried out by Affymetrix (now part of Thermo Fisher Scientific) using two closely related purpose-designed arrays. Among the available genome-wide genetic data of 488 377 participants, 49 950 participants were analysed using the UK BiLEVE Axiom array (Resource 149600), and the remaining 438 427 were assessed using the UKB Axiom array (Resource 149601), demonstrating a 95% marker content similarity between two arrays (ref: 10.1038/s41586-018-0579-z).

A total of 12 participants from the Samsung Medical Center and 413 participants registered in the UKB had the *APOE* ɛ2/ɛ4 genotype, who were excluded from the main analysis because of the putative opposing effects of ɛ2 and ɛ4 alleles.^25^ The *APOE* genotype was classified into three categories: ɛ2 carriers (ɛ2/ɛ2 and ɛ2/ɛ3), ɛ3 homozygotes (ɛ3/ɛ3) and ɛ4 carriers (ɛ3/ɛ4 and ɛ4/ɛ4).

### Measurement of vascular risk factors

Age, hypertension and diabetes were considered vascular risk factors. For individuals from the Samsung Medical Center, a health screening examination was conducted by a well-trained medical professional, following standardized protocols, as explained in a previous study.^30^ Assessment of hypertension and/or diabetes was based on the medical history of hypertension and/or diabetes or current medication usage, such as any antihypertensive and/or antidiabetic medication. For individuals registered in the UKB, their hypertension and diabetes states were determined through a combination of a touchscreen-based questionnaire, verbal interviews and linked hospital records, as outlined in our previous study.^30^

### Acquisition of brain MRI and image processing

All individuals from the Samsung Medical Center underwent a 3D volumetric brain MRI examination. 3D T_1_ turbo field echo MRI data were acquired using an Achieva 3.0-T MRI scanner (Philips, Best, the Netherlands) with the following imaging parameters: sagittal slice thickness, 1.0 mm with 50% overlap; no gap; repetition time of 9.9 ms; echo time of 4.6 ms; flip angle of 8; and matrix size of 240 × 240 px, resulting in 480 × 480 px image over a field view of 240 mm.

For individuals registered in the UKB, brain MRI scans were acquired at each of the three assigned sites using a 3.0-T Siemens Skyra MRI scanner. The scans included a T_1_-weighted sagittal 3D magnetization prepared rapid gradient echo image with the following imaging parameters: inversion time of 880 ms; repetition time of 2000 ms; voxel size of 1 × 1 × 1 mm^3^; matrix size of 208 × 256 × 256; and SENSE factor (R) of 2.0 (Miller, 2016: https://www.nature.com/articles/nn.4393).

The inner and outer cortical boundaries were reconstructed using T_1_-weighted MRI scans by the Montreal Neurological Institute CIVET pipeline (http://www.bic.mni.mcgill.ca/ServicesSoftware/CIVET). The cortical morphological measures, such as sulcal depth, cortical thickness and grey/white intensity ratio,^32^ were measured on the cortical surface at 81 924 vertices interconnected by 163 840 triangular edges. These metrics were subsequently re-sampled and mapped on to the surface template using the transformation derived from the surface registration to facilitate inter-subject comparisons.

### Graph convolutional neural network to predict the age of the entire brain and 11 different regions

**Figure 1:**
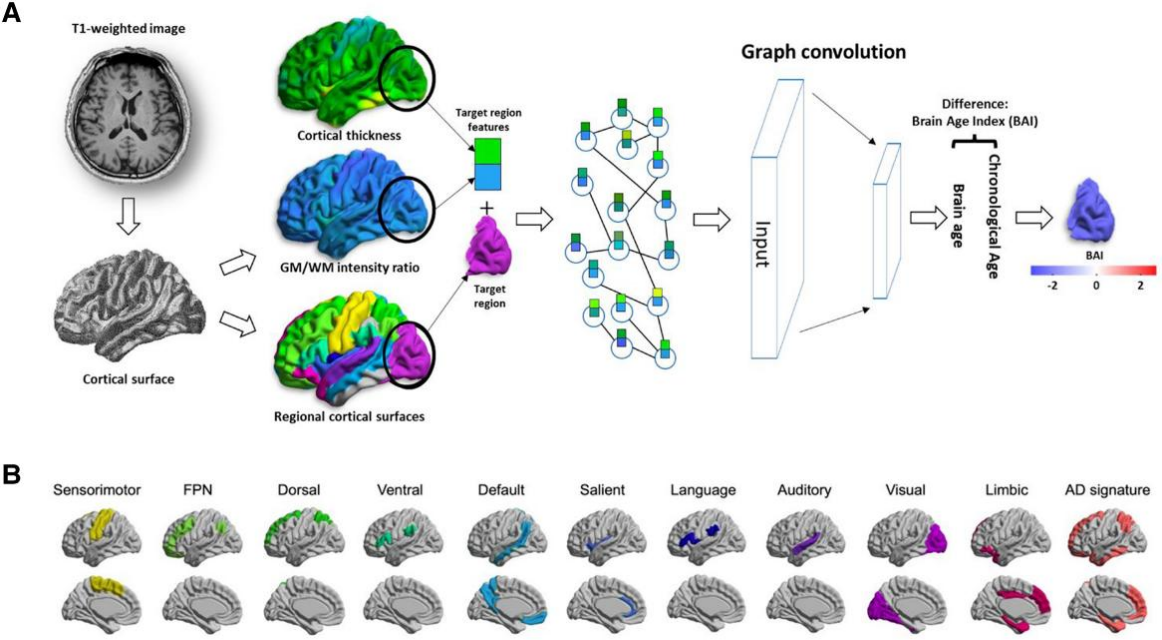
**GCN for regional brain age prediction.** (**A**) The input to the GCN was defined as a set of two feature vectors (i.e. cortical thickness and grey/white matter intensity ratio) in all vertices in a specific ROI and a sparse binary adjacency matrix representing the connections between each vertex and its neighbouring vertices. The output of the GCN model was a predicted brain age for each individual. The BAI was calculated as the difference between chronological age (age at scan) and predicted brain age, which represented the relative brain health status of the individual. (**B**) We used ROIs as 10 functional network regions in the cerebral cortex. These regions consisted of the sensorimotor, FPN, dorsal and ventral attention, default mode, salient, language and auditory, visual network and limbic cortical regions. In addition, we included the Alzheimer's disease signature region to assess whether brain aging in this region was associated with *APOE* ɛ4. To predict global brain age, the entire cortical surface was used as the ROI. AD, Alzheimer's disease; *APOE*, apolipoprotein E; BAI, brain age index; FPN, frontoparietal network; GCN, graph-based convolutional network; ROI, region of interest.

The proposed brain age model was based on the graph-based convolutional networks (GCNs) introduced in our previous study^33^ and was expanded for regional prediction (Fig. 1). The input to the GCNs was defined as a set of two feature vectors (cortical thickness and grey/white matter intensity ratio) at all vertices in a specified region of interest (ROI) and a sparse binary adjacency matrix representing the connections between each vertex and its neighbouring vertices. The output of the GCN model was the predicted brain age for each individual. The BAI was then calculated as the difference between the chronological age (age at scan) and the predicted brain age, which represents the relative brain health status of the individual.

To define the ROIs for which the BAIs were computed, the parcellation of the cortical surface into 65 cortical regions (ROIs) was performed using the AAL atlas. Yeo *et al*.'s atlas^34^ was used to merge the initial ROIs into 10 functional network regions in the cerebral cortex. These regions consisted of the frontoparietal network, dorsal attention, ventral attention, sensorimotor, default mode, salient, language, auditory and visual and limbic network regions. This atlas was adapted and labelled on the Montreal Neurological Institute cortical surface template. Additionally, we included the Alzheimer’s disease signature region to assess whether brain aging in this region was associated with *APOE* ɛ4. The Alzheimer’s disease signature region has been defined in a previous study.^35^ To predict global brain age, we used the entire cortical surface as the ROI.

### Statistical analyses

Student's *t*-test and *χ*^2^ tests were used to compare the continuous and categorical variables.

To assess whether *APOE* ɛ2 or ɛ4 is associated with increased BAI values among the Korean and UK populations, linear regression analyses were performed with the *APOE* genotype (ɛ2 carriers, ɛ4 carriers and ɛ3 homozygotes) as a predictor after controlling for age, sex, hypertension and diabetes in the Korean and UK populations. To assess whether the association between the *APOE* genotype and BAI differed based on ethnicity, linear regression analyses were performed by adding a two-way interaction term (*APOE* genotype × ethnicity) to both populations.

To evaluate the combined effects of *APOE* ɛ4 and vascular risk factors (age, hypertension or diabetes) on BAI among the Korean and UK populations, linear regression analyses were performed with *APOE* ɛ4 carriers [ɛ3 homozygotes (reference)] as a predictor and *APOE* ɛ4 carriers × age, *APOE* ɛ4 carriers × hypertension or *APOE* ɛ4 carriers × diabetes as a two-way interaction term while controlling for age, sex, hypertension and diabetes in the Korean and UK populations. To determine whether the combined effects of *APOE* ɛ4 and vascular risk factors differed among ethnic groups, linear regression analyses were performed by adding a three-way interaction term (*APOE* ɛ4 carriers × age × ethnicity, *APOE* ɛ4 carriers × hypertension × ethnicity or *APOE* ɛ4 carriers × diabetes × ethnicity) in both populations. Additionally, to identify the regions where the combined effects of *APOE* ɛ4 and vascular risk factors might differ among ethnic groups, linear regression analyses were performed with regional BAIs as outcomes by adding a three-way interaction term in both populations.

For all statistical analyses, we applied the false discovery rate (FDR) adjustment to correct for multiple comparisons. All reported *P*-values were two sided, with a significance threshold set at 0.05. All analyses were conducted using R version 4.3.0 (Institute for Statistics and Mathematics, Vienna, Austria; www.R-project.org).

## Results

### Demographics of CU populations in the UK and Korea

Table 1 presents the demographics of age- and sex-matched cohorts. The mean age did not differ between the Korean and UK populations (*P* = 0.441), and the female ratio did not differ between the two groups (*P* = 0.550). However, the proportion of *APOE* ɛ4 carriers was lower in the Korean population (19.3%) than that in the UK population (25.7%; *P* < 0.001). Compared with the UK population, the Korean population had a higher prevalence of hypertension (41.9% versus 31.8%, *P* < 0.001) and diabetes (18.4% versus 6.7%, *P* < 0.001).

**Table 1:** Demographics of the Korean and UK populations

	Korea (*n* = 2314)	UK (*n* = 6942)	*P*-value
Age, years	65.4 ± 6.8	65.6 ± 7.0	0.441
Sex (female)	1265 (54.7%)	3743 (53.9%)	0.550
*APOE* genotype			
ɛ2 carriers (ɛ2/ɛ2 and ɛ2/ɛ3)	268 (11.6%)	804 (11.6%)	
ɛ3 homozygotes (ɛ3/ɛ3)	1599 (69.1%)	4352 (62.7%)	
ɛ4 carriers (ɛ3/ɛ4 and ɛ4/ɛ4)	447 (19.3%)	1786 (25.7%)	
Hypertension	970 (41.9%)	2206 (31.8%)	<0.001
Diabetes	425 (18.4%)	414 (6.7%)	<0.001

Values are presented as mean ± standard deviation or number (%).

The *P*-values were obtained using independent *t*-tests and *χ*^2^ tests.

*APOE*, apolipoprotein E; UK, United Kingdom.

### Effect of *APOE* genotype on BAI

**Figure 2:**
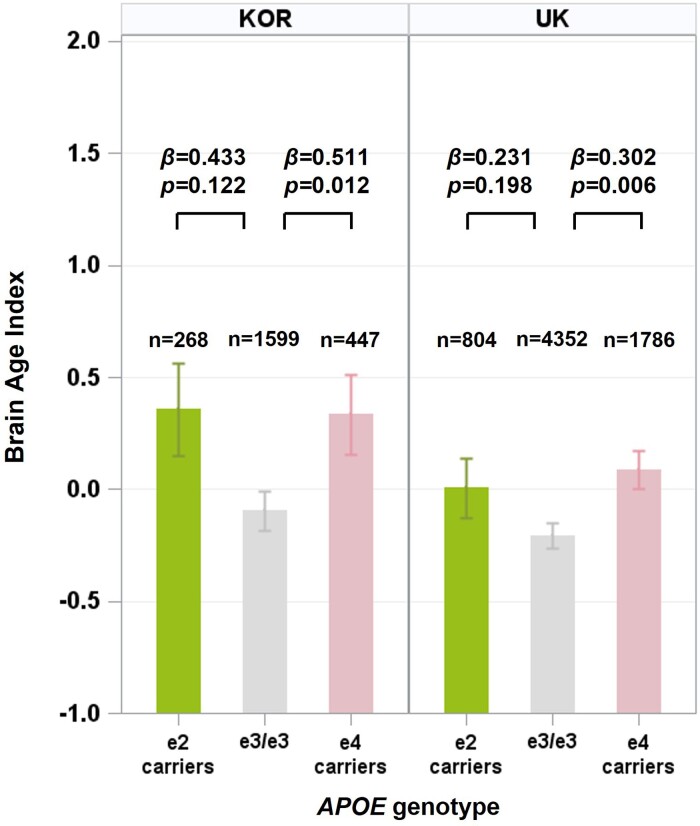
**BAI distribution in each *APOE* genotype subgroup in the Korean and UK populations.** The values depicted in the bar plot represent the mean BAI, and the values depicted in the error bar represent the standard error of the mean of each group. BAI = 0 indicates that the predicted brain age is equal to the chronological age, with higher values indicating an older-appearing brain than the chronological age. The *P*-values were obtained by linear regression analyses with *APOE* genotype (ɛ2 carriers, ɛ4 carriers and ɛ3 homozygotes) as a predictor after controlling for age, sex, hypertension and diabetes in the Korean and UK populations. The *P*-values for the interaction were obtained by linear regression analyses with the addition of a two-way interaction term (*APOE* genotype × ethnicity) to the covariates after controlling for age, sex, hypertension and diabetes in the Korean and UK populations. All *P*-values were modified after FDR correction for multiple comparisons. *N* represents the number of individuals used in the statistical analyses. *APOE*, apolipoprotein E; BAI, brain age index; FDR, false discovery rate; KOR, Korea; UK, United Kingdom.

BAI represents the difference between the chronological age from the predicted brain age (BAI = predicted brain age − chronological age). The *APOE* ɛ4 carriers in the Korean (*β* = 0.511, *P* = 0.012) and UK populations (*β* = 0.302, *P* = 0.006) showed increased BAI values compared with the *APOE* ɛ3 homozygotes (Fig. 2). There was no two-way interaction on BAI (*P* for ɛ4 × ethnicity = 0.405). In contrast, the *APOE* ɛ2 carriers in the Korean (*β* = 0.433, *P* = 0.122) and UK populations (*β* = 0.231, *P* = 0.198) did not show increased BAI values compared with the *APOE* ɛ3 homozygotes.

### Effect of *APOE* genotype and vascular risk factors on BAI

**Figure 3:**
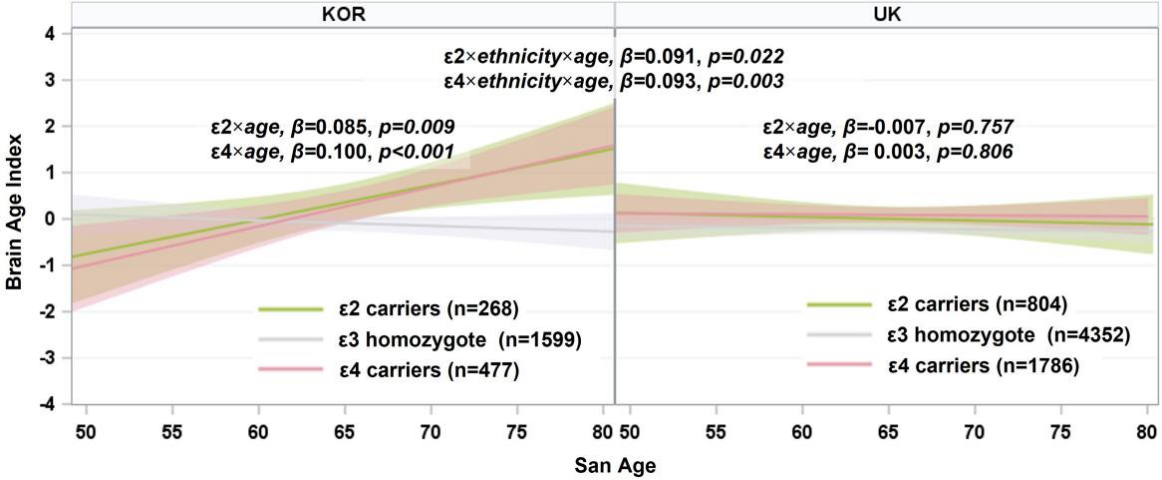
**Differential interaction effects between *APOE* genotype and age on BAI among ethnic groups.** BAI = 0 indicates that the chronological age at scan is the same as the predicted brain age, with higher values indicating an older-appearing brain than the chronological age. The *P*-values for two-way interaction were obtained by linear regression analyses with *APOE* ɛ4 or *APOE* ɛ2 carriers [ɛ3 homozygotes (reference)] as a predictor and the addition of each two-way interaction term (*APOE* ɛ4 carriers × age or *APOE* ɛ2 carriers × age) to the covariates after controlling for age, sex, hypertension and diabetes in the Korean and UK populations. The *P*-values for three-way interactions were obtained by linear regression analyses with the addition of each three-way interaction term (*APOE* ɛ4 carriers × age × ethnicity or *APOE* ɛ2 carriers × age × ethnicity) to the covariates after controlling for age, sex, hypertension and diabetes in the Korean and UK populations. All *P*-values were modified after FDR correction for multiple comparisons. *N* represents the number of individuals used in the statistical analyses. *APOE*, apolipoprotein E; BAI, brain age index; KOR, Korea; UK, United Kingdom.

In Koreans, the adverse effects of the *APOE* genotype on BAI increased with age (ɛ2 carriers × age, *β* = 0.085, *P* = 0.009; ɛ4 carriers × age, *β* = 0.100, *P* < 0.001; Fig. 3 and Table 2). However, in the UK population, no significant interaction was observed between *APOE* ɛ4 or *APOE* ɛ2 alleles and age in terms of BAI (ɛ2 carriers × age, *β* = −0.007, *P* = 0.757; ɛ4 carriers × age, *β* = 0.003, *P* = 0.806; Fig. 3 and Table 2). In fact, there was a three-way interaction in both *APOE* ɛ4 and *APOE* ɛ2 alleles, age and ethnicity on BAI (ɛ2 carriers × age × ethnicity, *β* = 0.091, *P* = 0.022; ɛ4 carriers × age × ethnicity, *β* = 0.093, *P* = 0.003; Fig. 3 and Table 2).

**Table 2:** Interaction effects of APOE genotype and vascular risk factors on BAI

Ethnicity	Interactions between *APOE* genotype and vascular risk factors	*β* (SE)	*P*-value
Korea	*APOE* ɛ4 × age	0.100 (0.027)	<0.001
	*APOE* ɛ4 × hypertension	0.777 (0.374)	0.038
	*APOE* ɛ4 × diabetes	0.612 (0.488)	0.209
	*APOE* ɛ2 × age	0.085 (0.033)	0.009
	*APOE* ɛ2 × hypertension	0.310 (0.473)	0.512
	*APOE* ɛ2 × diabetes	1.224 (0.577)	0.128
UK	*APOE* ɛ4 × age	0.003 (0.015)	0.806
	*APOE* ɛ4 × hypertension	−0.355 (0.218)	0.103
	*APOE* ɛ4 × diabetes	−0.485 (0.444)	0.274
	*APOE* ɛ2 × age	−0.007 (0.021)	0.757
	*APOE* ɛ2 × hypertension	0.096 (0.316)	0.760
	*APOE* ɛ2 × diabetes	0.5833 (0.584)	0.318
Both	*APOE* ɛ4 × ethnicity × age	0.093 (0.032)	0.003
	*APOE* ɛ4 × ethnicity × hypertension	1.091 (0.442)	0.014
	*APOE* ɛ2 × ethnicity × age	0.091 (0.040)	0.022
	*APOE* ɛ2 × ethnicity × hypertension	0.211 (0.581)	0.716

Two-way interactions were analysed using linear regression models with *APOE* ɛ4 or *APOE* ɛ2 carriers [ɛ3 homozygotes (reference)] as a predictor and by adding each two-way interaction term (*APOE* ɛ4 carriers × age, *APOE* ɛ4 carriers × hypertension, *APOE* ɛ4 carriers × diabetes, *APOE* ɛ2 carriers × age, *APOE* ɛ2 carriers × hypertension or *APOE* ɛ2 carriers × diabetes) to the covariates after controlling for age, sex, hypertension and diabetes in the Korean and UK populations.

Three-way interactions were analysed using linear regression models with the addition of each three-way interaction term (*APOE* ɛ4 carriers × age × ethnicity, *APOE* ɛ4 carriers × hypertension × ethnicity, *APOE* ɛ2 carriers × age × ethnicity or *APOE* ɛ2 carriers × hypertension × ethnicity) to the covariates after controlling for age, sex, hypertension and diabetes in the Korean and UK populations.

*APOE*, apolipoprotein E; BAI, brain age index; UK, United Kingdom.

**Figure 4:**
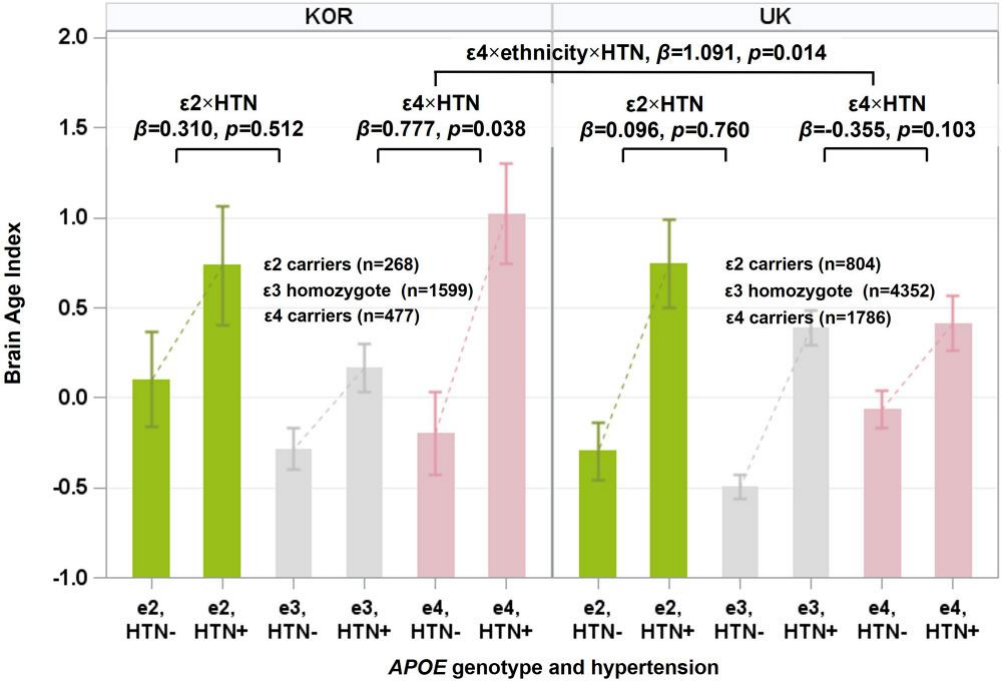
**Differential interaction effects between *APOE* genotype and hypertension on BAI among ethnic groups.** The values depicted in the bar plot represent the mean BAI, and the values depicted in the error bar represent the standard error of the mean of each group. BAI = 0 indicates that the chronological age at scan is the same as the predicted brain age, with higher values indicating an older-appearing brain than the chronological age. The *P*-values for two-way interaction were obtained by linear regression analyses with *APOE* ɛ4 or *APOE* ɛ2 carriers [ɛ3 homozygotes (reference)] as a predictor and the addition of each two-way interaction term (*APOE* ɛ4 carriers × hypertension or *APOE* ɛ2 carriers × hypertension) to the covariates after controlling for age, sex, hypertension and diabetes in the Korean and UK populations. The *P*-values for three-way interaction were obtained by linear regression analyses with the addition of each three-way interaction term (*APOE* ɛ4 carriers × hypertension × ethnicity or *APOE* ɛ2 carriers × hypertension × ethnicity) to the covariates after controlling for age, sex, hypertension and diabetes in the Korean and UK populations. *N* represents the number of individuals used in the statistical analyses. *APOE*, apolipoprotein E; BAI, brain age index; HTN, hypertension; KOR, Korea; UK, United Kingdom.

In the Korean population, *APOE* ɛ4 carriers and hypertension had a significant interaction with BAI (ɛ4 carriers × hypertension, *β* = 0.777, *P* = 0.038; Fig. 4 and Table 2), indicating that the effect of *APOE* ɛ4 on BAI was more pronounced in individuals with hypertension than in those without hypertension. However, no interaction was observed between *APOE* ɛ2 carriers and hypertension (ɛ2 carriers × hypertension, *β* = 0.310, *P* = 0.512). In the UK group, no interaction was observed between *APOE* ɛ4 or *APOE* ɛ2 carriers and hypertension in terms of the BAI (ɛ2 carriers × hypertension, *β* = 0.096, *P* = 0.760; ɛ4 carriers × hypertension, *β* = −0.355, *P* = 0.103). In fact, there was a three-way interaction on BAI (ɛ4 carriers × hypertension × ethnicity, *β* = 1.091, *P* = 0.014; Fig. 4 and Table 2).

*APOE* ɛ4 or *APOE* ɛ2 carriers and diabetes had no interaction effect on the BAI in the Korean (ɛ2 carriers × diabetes, *β* = 1.224, *P* = 0.128; ɛ4 carriers × diabetes, *β* = 0.612, *P* = 0.209), and UK (ɛ2 carriers × diabetes, *β* = 0.583, *P* = 0.318; ɛ4 carriers × diabetes, *β* = −0.485, *P* = 0.274) populations. However, the interaction effect between *APOE* ɛ4 or *APOE* ɛ2 carriers and diabetes showed a similar trend to those between *APOE* ɛ4 or *APOE* ɛ2 carriers and hypertension the Korean and UK groups.

**Figure 5:**
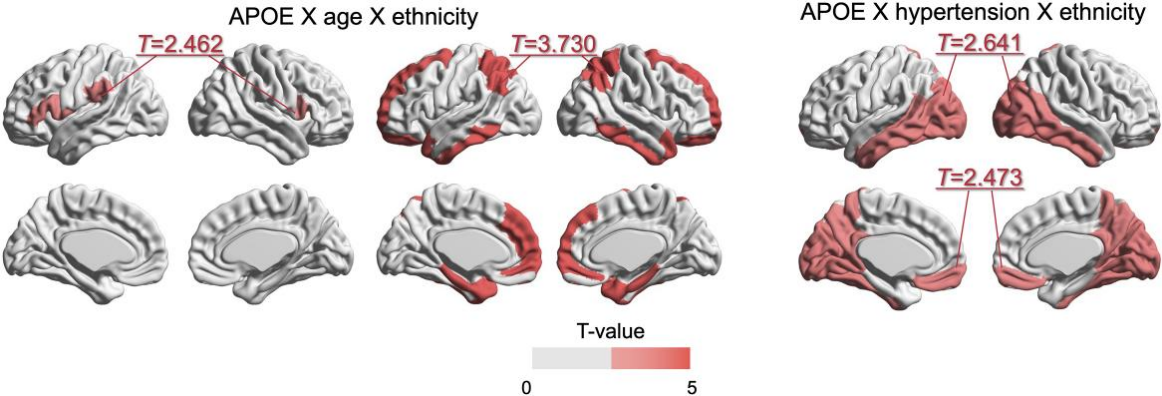
**Effects of the three-way interaction among *APOE* ɛ4, age or hypertension and ethnicity on regional brain age.**  *T*-value maps represent the effect size of the interactions (grey: not significant after FDR correction). The generated maps indicate that age and hypertension had more detrimental effects on regional brain aging in individuals when combined with *APOE* ɛ4, particularly in the Korean cohort than in the UK cohort. With regard to age, these effects were significant in the language and Alzheimer’s disease signature regions. The effects of hypertension were significant in the visual and limb regions. *APOE*, apolipoprotein E; KOR, Korea; UK, United Kingdom.

As illustrated in Fig. 5, there were interactions among *APOE* ɛ4 carriers, age and ethnicity with BAI in Alzheimer’s disease signature (*β* = 0.087, *P* = 0.011) and language network (*β* = 0.083, *P* = 0.014) regions and interactions among *APOE* ɛ4 carriers, age and hypertension with BAI in default (*β* = 1.177, *P* = 0.015) and visual network (*β* = 1.252, *P* = 0.009) regions.

## Discussion

In the present study, we conducted a systematic investigation to understand the effects of *APOE* ɛ4 allele on the BAI in relation to vascular risk factors, including age, hypertension and diabetes, in a large sample of CU Korean and UK populations. Our study yielded several key findings. First, both the Korean and UK populations demonstrated that *APOE* ɛ4 carriers showed an increased BAI compared with the *APOE* ɛ3 homozygotes, indicating a consistently greater deviation from the expected brain age across different ethnic groups. Second, the adverse effects of *APOE* ɛ4 on BAI increased with age in the Korean population, suggesting a cumulative impact on brain aging. In the UK population, the effect of *APOE* ɛ4 on BAI remained constant regardless of age. Lastly, the adverse effects of *APOE* ɛ4 on BAI were more pronounced in individuals with hypertension than in those without hypertension in the Korean population. In contrast, no significant interaction was observed between *APOE* ɛ4 and hypertension in the UK population. Overall, the combined effects of *APOE* ɛ4 and vascular risk factors, particularly age and hypertension, had a stronger influence on BAI in the Korean population than in the UK population. These findings underscore the need for ethnicity-specific strategies to manage vascular risk factors and mitigate *APOE* ɛ4-related brain aging in CU individuals.

Our first main finding indicated that *APOE* ɛ4 carriers showed an increased BAI compared with the *APOE* ɛ3 homozygotes in both Korean and UK populations. Only a few studies have investigated the relationship between *APOE* ɛ4 and BAI, and they have mainly focused on European and American populations.^19,20^ Considering that BAI is estimated based on brain atrophy, our findings align with those of previous studies conducted in Asian populations. Previous studies performed in Asian populations also demonstrated that *APOE* ɛ4 contributes to increased brain atrophy in the medial temporal, posterior cingulate and insular regions.^27,36^ These findings further support our observation of the effects of *APOE* ɛ4 on BAI in the Korean and UK populations.

Our second major finding was that the impact of *APOE* ɛ4 on BAI was reinforced by aging in the Korean population, whereas in the UK population, the effects remained constant, regardless of age. This difference in the combined effects of age and *APOE* ɛ4 on BAI between ethnic groups may be attributed to socioeconomic disparities and historical factors, such as poverty and food shortages, during the exploited colonial era of the Japanese Empire (1910–45) and the Korean War (1950–53).^37^ Furthermore, before 1960, a smaller number of students in Korea received secondary education or higher education than those in the UK.^37,38^ These difficult experiences may have had lasting effects on their brain health and made them more vulnerable to the combined effects of aging and *APOE* ɛ4. A previous study suggested that childhood stress contributes to epigenetic changes in miRNA levels associated with Alzheimer’s disease.^39^ Alternatively, the genetic differences between the Korean and UK populations could also play a role in the observed ethnic disparities. Previous studies from our group showed that variations in the gene encoding brain-derived neurotrophic factor are significantly associated with increased β-amyloid uptake in the brains of Korean individuals, but not in European individuals.^21^ Considering that polymorphisms in brain-derived neurotrophic factor may be associated with vulnerability in brain structure networks,^40^ these genetic differences might contribute to accelerated brain aging resulting from the combined effects of aging and *APOE* ɛ4 in the Korean population compared with the UK population. Moreover, the deleterious effect of *APOE* ɛ2 on BAI increases with age in the Korean population. Although the exact pathomechanism underlying this relationship is not fully understood, the association between *APOE* ɛ2 and vasculopathy might explain the combined effects of aging and *APOE* ɛ2.^41^ Furthermore, ɛ2 carriers had higher BAI values than those with ɛ3/ɛ3, but the difference was not significant. Our finding was consistent with that of a previous study that used measures of brain atrophy.^42^ Although *APOE* ɛ2 is a protective factor against the development of Alzheimer’s disease through its amyloid burden-lowering effect, a previous systematic review revealed that *APOE* ɛ2 may be a risk factor for brain atrophy rather than a protective factor.^42^

Finally, our study revealed an intriguing interaction between *APOE* ɛ4, hypertension and brain aging in the Korean population, which was not observed in the UK population. In the Korean population, the adverse effects of *APOE* ɛ4 on BAI were more pronounced in individuals with hypertension than in those without hypertension. These ethnic disparities in the combined effects of hypertension and *APOE* ɛ4 on BAI may be attributed to several factors. First, the effects of *APOE* ɛ4 and hypertension on brain health were more prominent in the Asian population than in the European population.^25,26,43^ For instance, hypertension is strongly associated with increased arterial stiffness^44^ and carotid intima thickness^45,46^ in Asians, which may further contribute to brain atrophy. In fact, interactions between hypertension-related arterial stiffness and *APOE* ɛ4 contribute to brain atrophy and cognitive decline.^47,48^ Additionally, studies have reported that the effects of *APOE* ɛ4 on the development of dementia are more prominent in Asians than in Europeans. Second, both *APOE* ɛ4 and hypertension are linked to the breakdown of the blood–brain barrier. Hypertension can lead to microvascular damage in the brain and disrupt the integrity of the blood–brain barrier. This disruption may impede the clearance of toxic substances associated with *APOE* ɛ4, such as amyloids, through impaired perivascular drainage, eventually resulting in increased brain atrophy and brain age.^49^ Therefore, the vulnerability of *APOE* ɛ4 and hypertension to brain health in Asian populations may underlie the observed ethnic differences in the combined effects of *APOE* ɛ4 and hypertension on the BAI. The intricate interplay between genetic factors, hypertension and brain aging requires further investigation to fully understand the underlying mechanisms and their implications for the provision of personalized healthcare and prevention of neurodegenerative diseases.

These interaction effects on the BAI were observed in specific functional brain network regions. Specifically, *APOE* ɛ4, age and ethnicity showed significant interaction effects on the regional BAI within the Alzheimer’s disease signature and language network regions. This finding aligns with those of previous studies, which indicated that *APOE* ɛ4-related brain atrophy is predominantly observed in areas associated with Alzheimer’s disease, such as the medial temporal and inferior parietal regions.^1,3^ These regions are vulnerable to the effects of *APOE* ɛ4. Additionally, *APOE* ɛ4, hypertension and ethnicity showed interaction effects on the regional BAI within the default and visual network regions. Previous studies have reported connectivity changes in the default mode network regions in individuals with preclinical Alzheimer’s disease and hypertension, which can eventually contribute to cognitive decline.^50^ Therefore, the combined effects of *APOE* ɛ4 and hypertension may have specific effects on these functional brain networks. However, the specific implications of these interaction effects on the BAI within the specific brain networks have not yet been extensively investigated. Further studies are warranted to comprehensively understand the clinical significance and implications of these findings.

### Limitations

The strengths of our study include the large sample size of the two cohorts and well-balanced clinical demographics between the two cohorts after propensity score matching. However, our study had several limitations. First, we did not consider Alzheimer’s disease-specific biomarkers, including β-amyloid and tau, which are well-known mediators of *APOE* ɛ4 allele and BAI. Second, although we performed propensity score matching, the differences in the prevalence of vascular risk factors between the two ethnic cohorts may be potential confounders. Third, the number of *APOE* ɛ2 carriers was relatively small to identify the association between *APOE* ɛ2 allele and BAI. Fourth, although we have found the effect of sex on BAI in our previous study,^30^ the present study was not conducted after stratifying by sex due to a relatively small number of several subgroups (e.g. female ɛ2 carriers with hypertension). Instead, we adjusted the sex as a confounder to mitigate the issue. Finally, our cross-sectional study did not determine the causal effects of *APOE* ɛ4 allele and age/hypertension on BAI. Therefore, future studies are necessary to identify the neurodegenerative trajectory in relation to *APOE* ɛ4 and the presence of other risk factors. Nevertheless, our study is noteworthy as our results highlighted that the ethnic difference in *APOE* ɛ4 effect becomes significant with age and the presence of hypertension.

## Conclusions

In conclusion, our study revealed ethnic differences in the combined effects of *APOE* ɛ4 genotype and vascular risk factors on brain age acceleration. These findings emphasize the need for ethnicity-specific strategies to mitigate accelerated brain aging in *APOE* ɛ4 carriers. Further research is needed to understand the underlying mechanisms and develop effective interventions to address accelerated brain aging in *APOE* ɛ4 carriers.

## Data Availability

We have shared the trained model that was used to predict brain age available on the GitHub repository (https://github.com/pks1207/regional_Brain_age). The data that support the findings of this study are available on request from the corresponding author.
